# Hybrid Adaptive Segmentation and Morphology-Based Classification of EOG for Automated Detection of Phasic and Tonic REM Sleep

**DOI:** 10.3390/s26041389

**Published:** 2026-02-23

**Authors:** Tomáš Nagy, Marek Piorecký, Karolína Janků, Václava Piorecká

**Affiliations:** 1 Faculty of Biomedical Engineering, Czech Technical University in Prague, 27201 Kladno, Czech Republic; 2 Sleep and Chronobiology Research Centre, National Institute of Mental Health, 25067 Klecany, Czech Republic; 3 Clinical Research Program, National Institute of Mental Health, 25067 Klecany, Czech Republic

**Keywords:** REM sleep, phasic REM, tonic REM, EOG, adaptive segmentation, wavelets, custom morphology kernel, MAD thresholding, Bayesian optimization, event fragmentation, polysomnography

## Abstract

Rapid eye movement (REM) sleep is increasingly understood as a heterogeneous state composed of two neurophysiologically distinct microstates: tonic REM and phasic REM. Phasic REM, defined by brief clusters of saccadic eye movements and transient cortical activation, has been linked to emotional memory consolidation, sensorimotor integration, and autonomic modulation. Despite its importance, automated quantification of phasic versus tonic REM remains uncommon, mainly because existing electrooculography (EOG) methods rely on fixed thresholds or generic wavelet families that do not accurately capture real saccade morphology in clinical polysomnography (PSG). This study introduces a fully automated framework for detecting phasic REM based on hybrid adaptive segmentation of a single EOG channel. The segmentation algorithm fuses median absolute deviation (MAD) amplitude-change detection with a morphology score derived from a custom saccade kernel built from manually verified EyeCon recordings. Segment boundaries are refined using local derivative extrema to improve temporal alignment. A supervised support vector machine (SVM) classifier further refines segment labels using features based on saccade morphology, including correlations with custom log-sigmoid templates and a morphology similarity measure. All segmentation and classification hyperparameters were optimized exclusively on controlled EyeCon datasets with precise ground-truth event markers. The final model was then applied without modification to 21 full-night clinical PSG recordings. Event-level analysis on EyeCon yielded 92.9% correct detections, with 5.3% fragmentation and 1.8% missed events. When aggregated into saccadic bursts, the resulting REM microstructure was physiologically consistent: phasic REM accounted for 31.8 ± 3.5% of REM duration, and tonic REM for 68.2 ± 3.5%. Additional EEG analysis confirmed increased beta and gamma power during phasic REM, supporting physiological validity. The proposed framework provides an interpretable, morphology-aware, and computationally efficient tool for large-scale REM microstructure research. Its single-channel design and external validation on clinical PSG recordings make it suitable for both retrospective analyses and future clinical applications.

## 1. Introduction

Rapid eye movement (REM) sleep is no longer viewed as a homogeneous physiological state but rather as a dynamic alternation between two distinct microstates: tonic REM and phasic REM [1,2]. Tonic REM is characterized by relative ocular quiescence, stable autonomic activity, and sustained cortical desynchronization [3,4]. In contrast, phasic REM consists of transient bursts of rapid saccadic eye movements accompanied by increased cortical excitability, limbic activation, and autonomic fluctuations [1,5]. The role of the phasic portion of REM is thought to support emotional memory consolidation, sensorimotor integration, affective processing, and dream generation [6,7].

Despite its physiological importance, REM microstructure remains almost entirely unannotated in clinical practice. Current polysomnography (PSG) scoring guidelines, including the AASM manual [8], do not require distinguishing between tonic and phasic REM [1]. Given the absence of such scoring requirements, PSG assessors rarely annotate and analyze these microstructures manually [9]. As no validated manual or portable automated system exists for phasic/tonic REM classification, systematic evaluation of REM microdynamics has remained uncommon [10].

Existing methods for identifying the phasic component of REM rely primarily on detecting rapid eye movements from electrooculography (EOG) [9]. The number and placement of EOG channels in PSG varies across laboratories, typically emphasizing the horizontal axis while giving minimal attention to the vertical component [2]. Simple time-domain thresholding based on amplitude or derivative has historically been used to detect saccades [11,12], but these approaches are highly sensitive to drift, inter-subject variability, fitting differences, and observational noise.

Wavelet-based approaches, including Haar and Daubechies (DB4), have shown improved robustness for transient detection in EOG and PSG [13,14,15]. However, these methods rely on analytic wavelet shapes that do not fully reflect the morphology of real saccadic eye movements in clinical recordings. Furthermore, most prior studies have focused on isolated saccade or blink detection rather than reconstructing the full microstructure of REM sleep on the basis of burst dynamics.

To address these limitations—including variability in EOG montage configurations and the lack of a standardized manual—we developed a fully automated, morphology-aware, and temporally precise system for reconstructing tonic and phasic REM from a single EOG channel. To overcome the shortcomings of existing detectors, we propose a hybrid adaptive segmentation framework that integrates (i) a median absolute deviation (MAD) amplitude-change metric [12] with (ii) a morphology score computed using a custom saccade-derived kernel constructed from manually verified eye-movement events. Unlike generic wavelets, this kernel captures characteristic rise, plateau, and fall components of real saccades observed in PSG-grade EOG, enabling more physiologically grounded detection.

Several groups have previously attempted to automate the analysis of eye movements in REM sleep using EOG. Early work focused on time-domain criteria: Gopal and Haddad introduced a slope-plus-amplitude detector achieving high correlation with visually scored REM counts in overnight PSG [11]. Boukadoum et al. refined these methods, comparing amplitude-, slope-, and template-based detectors and demonstrating the importance of adaptive thresholds and preprocessing [12,16]. Although such techniques showed that automatic REM detection from EOG is feasible, they typically counted eye movements per epoch rather than producing temporally precise segmentation.

Wavelet-based detectors were later introduced to better capture transient morphologies. Tsuji et al. applied multiscale wavelet energy analysis to identify REMs in narcolepsy [14]. Juhász et al. used wavelets to study REM microstructure, focusing mainly on short phasic events [15]. Pettersson et al. showed that Haar wavelets can effectively localize blink-like transients in general EOG analysis [13]. These studies confirmed the value of wavelet analysis but relied on generic shapes that may not optimally match saccadic morphology in PSG.

Outside sleep research, EOG-based classifiers have been extensively explored for human–computer interaction and activity recognition. Bulling et al. combined time- and frequency-domain features with machine-learning classifiers for classifying saccades, blinks, and fixations during daily activities, achieving accuracies above 90% in controlled settings [17]. Toivanen et al. designed a real-time probabilistic algorithm using amplitude and velocity features to detect eye-movement categories [18]. Other studies employing SVMs, k-NN, and neural networks report similarly high accuracies in controlled environments [19,20]. These approaches, however, were not designed for REM sleep microstructure and do not provide the temporally precise segmentation needed for burst-level phasic classification.

In contrast, only a few studies have explicitly targeted REM microstructure or tonic/phasic characterization using biosignals. Recent work has addressed related goals using EEG/EOG in sleep-stage classification or disorder detection pipelines [21,22,23,24]. However, these approaches typically focus on (i) coarse sleep staging rather than continuous REM microstate reconstruction, (ii) feature representations not explicitly constrained by saccade morphology, and/or (iii) multi-modal PSG analytics where EOG is not the primary driver. Accordingly, a gap remains for an interpretable, EOG-driven framework that reconstructs tonic and phasic REM intervals from saccade burst dynamics with event-level, morphology-aware detection. Most clinical work still relies on visual inspection of REM density or cluster-like events [10]. Wavelet-based phasic detection studies typically use EEG rather than EOG [1,15] and often stop short of reconstructing full-night tonic/phasic intervals directly from eye-movement dynamics.

The present work addresses this gap by combining hybrid adaptive segmentation with a lightweight morphology-aware classifier trained solely on EyeCon ground-truth data [25]. Segmentation hyperparameters were optimized to reward accurate onset/offset localization and to penalize fragmentation or merging. A supervised support vector machine refines segment labels using features derived from saccade morphology, including correlation with a custom kernel and amplitude–duration characteristics, without relying on velocity-based thresholds or multichannel voting. The optimized system is applied unchanged to 21 full-night PSG recordings, yielding physiologically plausible tonic/phasic REM proportions consistent with established ranges [1,15]. This approach bridges the gap between earlier EOG-based REM detectors and the need for scalable, reproducible REM microstructure quantification.

### A Priori Hypotheses

Before conducting the evaluation, we formulated the following a priori hypotheses. First, we hypothesized that a hybrid detector combining a robust amplitude-change metric with morphology-aware template matching would yield higher event-level detection accuracy and lower fragmentation than fixed-threshold or generic wavelet-based EOG methods.

Second, we hypothesized that segmentation and classification parameters optimized exclusively on externally labeled EyeCon recordings would generalize to unseen full-night clinical PSG data without dataset-specific recalibration.

Third, we hypothesized that phasic REM intervals reconstructed from detected saccade bursts would exhibit physiologically plausible EEG signatures, and specifically increased low-beta and gamma power compared to tonic REM.

## 2. Materials and Methods

**Figure 1:**
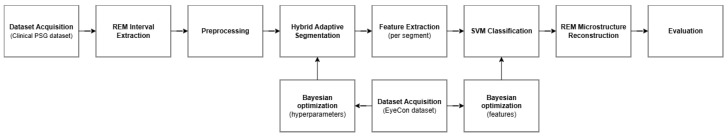
Block diagram of the proposed processing pipeline. The diagram illustrates each stage of the workflow in the order in which they are described in this section.

This chapter is organized to follow the workflow of the proposed signal-processing pipeline. Each subsection describes one stage of the method, presented in the same order in which the algorithm operates, from data acquisition to the derivation of phasic and tonic REM structure. A summary of the proposed processing pipeline is provided in Figure 1.

### 2.1. Datasets

This study used two complementary datasets originating from independent clinical and experimental settings, recorded on different acquisition systems and scored by different experts. This diversity ensured that the proposed methodology was evaluated on data with distinct signal characteristics, noise profiles, and recording conditions.

#### 2.1.1. EyeCon Ground-Truth Dataset

The EyeCon dataset (2020–2023, University of Malta) consists of high-quality experimental recordings specifically designed for precise eye-movement research [25]. Each session contains horizontal and vertical periocular EOG channels sampled at 256 Hz, accompanied by millisecond-accurate event markers transmitted via LabStreamingLayer. These markers were generated using a synchronized stimulus presentation system and provide reliable timing for a wide range of elicited saccadic eye movements. The recordings include both reflexive and voluntary saccades captured under controlled lighting and head-stabilized conditions, resulting in a clean and well-characterized reference dataset suitable for methodological development.

#### 2.1.2. Clinical PSG Dataset

The clinical dataset comprises 21 full-night polysomnography (PSG) recordings obtained from healthy adult participants (mean age = 23.42 ± 2.92, 15 men, 16 women). All recordings were scored manually by trained sleep technicians according to AASM guidelines [26]. The PSG montage included two periocular EOG channels—EOG1 positioned above the right eye and EOG2 lateral to the left eye—sampled at 250 Hz. These recordings reflect typical clinical variability encountered in overnight sleep studies, including slow drifts, non-ocular physiological artifacts, and changes in electrode impedance across the night. Sleep staging annotations allow precise isolation of REM intervals, during which most analyses in this study were conducted.

### 2.2. Preprocessing

All analyses were restricted to expert-scored REM intervals. Within each REM interval, the EOG1 channel underwent a standardized preprocessing pipeline consisting of baseline drift removal using a 1.5 s moving-average filter, followed by zero-phase 2nd-order Butterworth band-pass filtering in the 1–10 Hz band [27]. After filtering, the signal was normalized using a z-score transformation computed separately for each expert-scored REM interval. For each REM interval, the reference mean $\mu_{\mathrm{REM}}$ and standard deviation $\sigma_{\mathrm{REM}}$ were estimated exclusively from samples within that same interval, without using information from non-REM epochs. The normalized signal was defined as

(1) $$x_{\mathrm{norm}}\left(t\right)=\frac{x(t)-\mu_{\mathrm{REM}}}{\sigma_{\mathrm{REM}}},$$

where x(t) denotes the preprocessed EOG signal at time *t*.

REM-interval-based normalization reduces inter-subject amplitude variability while preserving within-REM temporal dynamics and has been used in prior EOG-based sleep and eye-movement analyses to improve cross-subject comparability [15,17,23].

All segmentation and classification procedures described in the following sections were applied exclusively to this preprocessed and normalized EOG1 signal, without using EEG or EMG information for detection or classification.

### 2.3. Hybrid Adaptive Segmentation

Segmentation was performed using a hybrid scoring framework that combines two complementary sliding-window measures: a robust amplitude-change score and a morphology score derived from correlation with a custom saccade template. Both scores were normalized within each REM interval to ensure consistency across subjects and recordings [27].

#### 2.3.1. Amplitude-Based Score (MAD Derivative)

The amplitude-change score is based on the absolute first derivative of the EOG signal,

(2) d(t)=|x(t)−x(t−1)|,

which quantifies rapid frame-to-frame voltage changes. High values of d(t) are typically associated with fast saccadic transitions, whereas low values reflect slow drift, baseline fluctuations, or stable regions without eye movements [12].

To obtain a threshold that is robust to outliers and inter-subject variability, the derivative was scaled using the median absolute deviation (MAD). The amplitude threshold was defined as

(3) $$T_{\mathrm{amp}}=k_{\mathrm{MAD}}\cdot \mathrm{MAD}\left(d\left(t\right)\right),$$

where MAD(d(t)) denotes the median absolute deviation of the derivative signal and $k_{\mathrm{MAD}}$ is a sensitivity parameter. This approach has been shown to be effective for saccade detection in EOG signals due to its robustness [12,13]. Samples for which $d\left(t\right)>T_{\mathrm{amp}}$ were treated as high-amplitude transitions and contributed to the amplitude score $S_{\mathrm{amp}}\left(t\right)$. This choice of MAD-based thresholding follows established recommendations for robust outlier-resistant normalization in biomedical signal processing [28].

#### 2.3.2. Morphology Score Using a Custom Saccade Kernel

To capture the characteristic morphology of real saccadic eye movements, a custom saccade kernel was constructed instead of relying on analytic wavelets (e.g., Haar, Daubechies), whose symmetry and lack of plateau structure do not reflect physiological saccade dynamics [14,15]. The kernel was derived from hundreds of manually verified saccades taken from both the EyeCon and PSG datasets. Each saccade was resampled to a common duration, normalized in amplitude, and aligned around its central peak to ensure consistent temporal structure. The aligned waveforms were then averaged to produce a representative prototype, which was subsequently L2-normalized to unit energy.

##### A Priori Validation of the Custom Saccade Kernel

**Figure 2:**
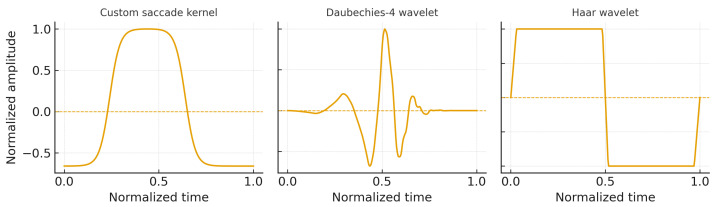
Comparison of the custom saccade-derived kernel (**left**) with Haar and DB4 wavelets (**middle**/**right**). The custom kernel captures the rising flank, plateau, and deceleration characteristic of real saccades, whereas analytic wavelets lack these morphology-specific features.

The custom kernel was designed as a physiology-informed prototype to reflect the characteristic multi-phase morphology of saccadic eye movements observed in PSG-grade EOG recordings. Its shape was qualitatively compared with commonly used analytic wavelets, including Haar and Daubechies-4 (DB4), highlighting the presence of an asymmetric rise, a short plateau, and a deceleration phase that are not explicitly encoded in generic wavelet bases (Figure 2).

Although a systematic numerical benchmark across multiple kernel families was not a primary aim of this study, the externally labeled EyeCon dataset was used during method development to verify that the proposed kernel reliably matches ground-truth saccade morphologies under controlled conditions. A more extensive quantitative comparison with alternative kernels and learned templates represents a natural direction for future work. The resulting kernel $h_{\mathrm{sac}}\left(t\right)$ reproduces the canonical three-phase saccade morphology: a rapid rising phase, a short plateau, and a deceleration phase. This physiologically informed shape stands in contrast to standard wavelets, as illustrated in Figure 2.

The morphology score was computed as the maximum absolute correlation between the preprocessed EOG signal and the saccade kernel within a sliding window:

(4) $$S_{\mathrm{morph}}\left(t\right)=\max_{\tau \in T}\left|\sum_{k=0}^{L-1}x\left(t+k+\tau \right)\;h_{\mathrm{sac}}\left(k\right)\right|.$$

Here, the summation evaluates the similarity between the signal segment x(t+k+τ) and the template $h_{\mathrm{sac}}\left(k\right)$ over all permissible temporal shifts τ∈T. The maximum absolute correlation provides a shape-sensitive score that is high whenever the local waveform matches the characteristic saccade morphology [27].

#### 2.3.3. Hybrid Score Fusion and Boundary Detection

To combine the complementary information provided by the amplitude-change and morphology-based measures, the two scores were fused into a single decision metric. The hybrid score was defined as

(5) $$S\left(t\right)=\alpha \;S_{\mathrm{amp}}\left(t\right)+\left(1-\alpha \right)\;S_{\mathrm{morph}}\left(t\right),$$

where α denotes the weighting factor controlling the relative influence of amplitude- and shape-based evidence. Higher values of α bias the detector toward rapid voltage transitions, whereas lower values favor morphological similarity to the saccade template. This approach draws on classical score-level fusion techniques in pattern recognition and neurophysiological signal analysis [27,29]. The parameter α was tuned using EyeCon ground-truth data to achieve an optimal balance between sensitivity and specificity.

Within each REM interval, segment boundaries were initially identified at time points where the hybrid score exceeded its REM-level mean by more than three standard deviations. This deviation-based criterion ensured high sensitivity to abrupt, localized changes in ocular activity while maintaining robustness to baseline fluctuations. The resulting set of boundary candidates yielded an initial segmentation with full temporal coverage of each REM interval.

#### 2.3.4. Boundary Refinement

The initial boundaries were subsequently refined to ensure temporal coherence and physiological plausibility of the detected segments. First, segments with a duration shorter than a predefined minimum duration min_dur were discarded, as such intervals typically reflect noise or sub-threshold fluctuations rather than meaningful eye movements. Second, segments separated by a temporal gap shorter than the merging threshold *g* were merged, preventing spurious fragmentation of what functionally constitutes a single movement. Finally, boundary positions were adjusted using local extrema of the EOG derivative to align segment edges with the nearest physiologically meaningful transitions, improving temporal precision [13,16].

The key hyperparameters and implementation settings used in the proposed segmentation and classification framework are summarized in Table 1.

### 2.4. Segment Classification Using SVM

Each segment produced by the hybrid adaptive segmentation was classified using a supervised support vector machine (SVM) with an RBF kernel [30]. All features were computed directly from the preprocessed EOG1 signal. The classifier was implemented using the LIBSVM library [31], and its formulation follows the standard theory of kernel-based learning [32].

Each segment is represented by a two-dimensional feature vector. The first feature was the amplitude–duration ratio, capturing the main-sequence relationship that links saccade amplitude to its temporal extent and providing a compact descriptor of event scale [33]. The second feature was derived from the maximum correlation between the segment waveform and a pair of log-sigmoid templates corresponding to rising and falling saccadic profiles, a method inspired by morphology-based classifiers used in eye-movement analysis [18].

For each segment, both template correlations were evaluated, and the higher of the two values was used as the final morphology-based feature. This approach preserves the directional sensitivity of the templates while maintaining a parsimonious feature space for classification [17].

Because the correlation is computed with two direction-specific templates, the method inherently allows for the discrimination of rising versus falling saccades, should such directional information be needed in downstream analyses. In the current work, however, only the magnitude of the better-matching correlation was used as the second classification feature.

Together, the main-sequence descriptor and the morphology-based correlation metric provide a concise and physiologically meaningful representation of each candidate eye-movement segment.

**Figure 3:**
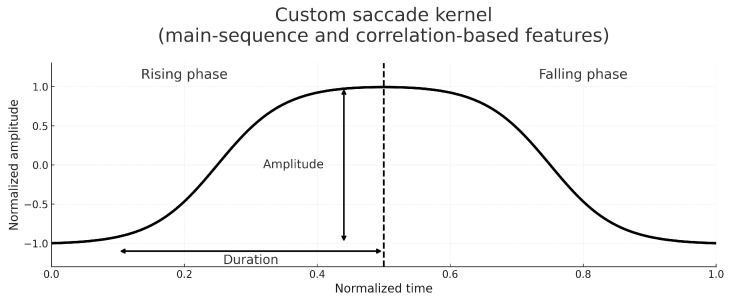
Custom saccade kernel illustrating the features used for segment classification. The amplitude and duration correspond to the main-sequence feature, while the rising and falling phases match the direction-specific correlation templates used in the feature vector.

The feature extraction and the morphology templates used for segment classification are illustrated in Figure 3.

Segments described by these two features were randomly divided into training and validation sets in a 2.33:1 ratio, resulting in two balanced datasets:

- Training: 894 segments (298 per class),
- Validation: 384 segments (128 per class).

This stratified sampling ensured equal representation of the three classes—*saccade*, *blink*, and *artifact*—across both sets, following standard procedures for balanced classification in small medical datasets. Although no synthetic oversampling was applied in this study, class-balancing techniques such as SMOTE are commonly used in biomedical machine-learning pipelines to address class imbalance [34].

The classifier operated with three classes, and only segments classified as *saccade* were subsequently used for reconstructing phasic REM microstructure. Classification performance was assessed using precision, recall, specificity, F1-score, and Cohen’s κ, providing a comprehensive evaluation of both class-wise accuracy and inter-method agreement [35].

### 2.5. Phasic and Tonic REM Classification

After obtaining segment-level classifications, all segments labeled as saccade were first ordered according to their temporal position. Consecutive saccadic segments were then grouped to form a saccadic burst. To ensure temporal continuity and account for short interruptions, bursts were further merged whenever they were separated by two or fewer non-saccadic segments, a method consistent with prior work on burst-based REM detection [1,15].

Phasic REM was defined as the union of all detected saccadic bursts, corresponding to periods of heightened ocular and cortical activity [5,6]. Tonic REM was defined as the complement of phasic REM within each annotated REM interval, reflecting more stable neurophysiological states.

The durations of phasic and tonic REM, their relative proportions, and the resulting phasic-to-tonic ratios were computed for each subject, following standard quantification strategies for REM microstructure [1,10].

### 2.6. Evaluation Metrics

EyeCon and PSG differ in signal montage and quality, and the labels were provided by different experts. For this reason, the evaluation was conducted separately for these two datasets.

For EyeCon (Training/Validation), performance was assessed using temporal segmentation accuracy, defined as onset and offset deviations within ±20 ms. Additional error modes, including fragmentation (split events) and merging (multiple events combined), were quantified. Standard classification metrics—precision, recall, specificity, and F1-score—were also computed, along with Cohen’s κ, which was used to assess inter-method agreement [35].

For PSG (Testing), which was originally used as no-ground-truth annotations for saccades, the evaluation focused on higher-level REM-structure characteristics. Specifically, we compared phasic and tonic REM proportions, assessed subject-level consistency, and examined the physiological plausibility of the obtained patterns relative to the established literature [1,15]. Experts also randomly validated automatically scored saccades.

### 2.7. Dataset Utilization Across the Processing Pipeline

A concise summary of how each dataset contributed to the individual components of the proposed processing pipeline is provided in Table 2. This overview clarifies which stages rely on ground-truth event annotations (EyeCon) and which analyses are performed exclusively on clinical PSG data.

## 3. Results

This section presents the performance of the proposed hybrid adaptive segmentation and SVM-based saccade classification framework. Results are reported for: (i) segmentation and classification accuracy on the EyeCon ground-truth dataset, and (ii) the phasic–tonic REM microstructure derived from full-night clinical PSG recordings. All PSG analyses were restricted to expert-scored REM intervals.

### 3.1. Segmentation Performance on EyeCon

Segmentation was evaluated by comparing automatically detected segments with ground-truth saccadic events using a temporal tolerance of ±20 ms. A detection was considered correct when one predicted segment overlapped exactly one ground-truth event. Fragmentation, missed events, and detected-to-ground-truth ratios were computed for each recording.

The hybrid segmentation achieved a high correct-detection rate (92.9%) while keeping both fragmentation and miss rates low (Table 3). The detected-to-ground-truth ratio (1.59) reflects mild over-segmentation, which was expected because the optimization objective penalized fragmentation more strongly than over-detection.

### 3.2. Saccade Classification Performance (SVM)

**Figure 4:**
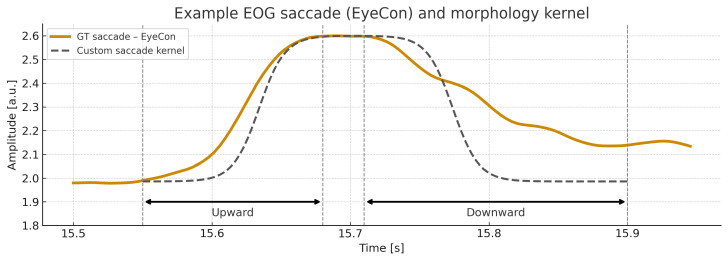
Example manually annotated saccade (EyeCon) overlaid with the morphology kernel used in the proposed segmentation framework. The ground-truth EyeCon saccade (solid trace) consists of an upward and downward phase, highlighted by horizontal arrows. Vertical dashed lines mark the expert-annotated saccade boundaries. The custom morphology kernel (dashed trace) is amplitude-scaled and time-aligned to the event, illustrating the morphological similarity exploited by the classifier.

Classification was performed exclusively using the SVM-based segment classifier. Predictions were evaluated with precision, recall, specificity, F1-score, and Cohen’s κ. A predicted saccade was considered correct when it overlapped a ground-truth event by at least 20 ms. An example of a manually annotated EyeCon saccade overlaid with the custom morphology kernel is shown in Figure 4.

Most classification errors arose from short-duration, low-amplitude saccades near blink boundaries. Despite this challenge, the SVM classifier provided stable and highly specific saccade labeling without requiring velocity-based detectors.

Table 4 shows the results of saccade classification on the EyeCon dataset. Precision reflects that the model correctly identified most positive examples. Recall indicates that the model was able to detect most true positives, despite some false negatives. High specificity means that the model discriminates very well between negative and positive cases. The F1-score shows a good balance between precision and recall. Cohen’s κ of 0.76±0.04 demonstrates substantial agreement between the classifier and expert scoring. Overall, the results show that the model has high performance in saccade classification, with a robust balance between accuracy and sensitivity.

### 3.3. REM Microstructure in Clinical PSG

After training exclusively on EyeCon, the model was applied to 21 full-night PSG recordings containing a priori no-ground-truth saccade labels. Within each REM interval, saccadic segments produced by the SVM classifier were grouped into *bursts*. Phasic REM was defined as the union of all bursts; the remainder was labeled tonic.

These results from Table 5 indicate that in this cohort of subjects, tonic REM sleep was significantly longer and accounted for a greater proportion of total REM sleep compared to phasic REM. The phasic to tonic REM ratio (0.471) suggests that tonic REM is present for a greater proportion of sleep time, which is consistent with expectations for healthy adult subjects. The inter-subject variability (SD) indicates that there is some variation among study participants, particularly in the duration of tonic REM sleep. Phasic REM accounted for approximately one-third of all REM sleep, consistent with previously reported ranges (20–35% in healthy adults). Inter-subject variability was low (SD ≈ 3.5%), indicating stable microstructure quantification across individuals. Across the 21 participants, the estimated proportion of phasic REM showed only moderate dispersion around the group mean (31.8%), with no extreme outliers. This pattern supports stable subject-level generalization of the proposed framework across the clinical PSG cohort.

### 3.4. EEG Differences Between Phasic and Tonic REM

Although EEG signals were not used for segmentation or classification, they were analyzed to provide an independent physiological validation of the reconstructed REM microstructure. Spectral power was compared between automatically identified phasic and tonic REM intervals using multitaper spectral analysis.

Phasic REM was associated with increased power in higher-frequency bands, specifically in the low-beta (13–20 Hz) and gamma (30–45 Hz) ranges, whereas delta and theta power were relatively reduced compared to tonic REM. This spectral pattern is consistent with prior reports linking phasic REM to transient cortical activation and increased arousal-like dynamics.

Importantly, these EEG differences were observed without using EEG information for detection, supporting the notion that the EOG-based reconstruction of phasic and tonic REM captures physiologically meaningful microstate differences rather than reflecting algorithmic bias.

### 3.5. Summary of Quantitative Outcomes

The combined segmentation and SVM classification framework achieved:

- 92.9% correct event detection;
- 5.3% fragmentation rate;
- 1.8% miss rate;
- Highly accurate saccade classification (F1-score = 0.900);
- Physiologically plausible REM microstructure (phasic ≈32%).

Together, these results demonstrate that morphology-aware segmentation combined with SVM classification enables reliable and interpretable reconstruction of the REM microstructure from single-channel EOG data at the least approximate horizontal montage.

## 4. Discussion

This study introduces a fully automated and morphology-aware framework for detecting saccadic eye movements and reconstructing phasic–tonic REM microstructure from a single clinical-grade EOG channel. In contrast to earlier approaches relying on fixed thresholds, velocity criteria, or general-purpose analytic wavelets, the proposed system integrates a robust amplitude-change metric with a custom morphology kernel derived from manually verified saccades. This kernel reproduces the characteristic asymmetric rise, brief plateau, and deceleration phases of real ocular movements recorded in PSG. Combined with a compact SVM classifier trained exclusively on the controlled EyeCon dataset, the method achieves high temporal precision and demonstrates strong generalizability to full-night clinical polysomnography without requiring dataset-specific recalibration.

### 4.1. Interpretation of Segmentation Performance

The hybrid adaptive segmentation achieved a correct event-detection rate of 92.9%, with only 1.8% missed events and 5.3% fragmented events across all overnight PSG recordings. These results indicate that the method reliably delineates saccadic events despite substantial variability in amplitude, drift, and noise inherent to nocturnal EOG. The detected/ground-truth ratio of 1.59 reflects a deliberate design choice: the segmentation prioritizes capturing each physiological event as a complete, uninterrupted segment. This choice is essential, as the resulting segments serve directly as inputs to the morphology-based SVM classifier. Fragmentation would disrupt amplitude–duration measures and degrade the stability of morphology-dependent features.

Classical pointwise metrics (samplewise TP/FP/FN) were not used because they penalize extended segment boundaries even when these boundaries correctly capture the full morphology of a saccade. In this context, event-level metrics—onset and offset accuracy, fragmentation rate, missed-event rate, and detected/ground-truth ratio—provide a more meaningful evaluation of segmentation performance, directly reflecting whether each event is captured as a single physiologically coherent unit.

A key contributor to segmentation performance is the morphology kernel constructed from real saccades. Unlike analytic wavelets such as Haar or Daubechies-4, which are frequently used for transient detection in biomedical signals due to their compact support and multi-resolution properties [27], the custom kernel is tailored to the asymmetric shape of ocular movements in PSG. This physiologically informed design markedly improves discrimination between true saccades and non-saccadic transients such as blink-induced slopes, slow drifts, motion artifacts, and impedance fluctuations.

Earlier approaches for automatic REM-related eye-movement detection were dominated by simple amplitude or slope thresholding applied to prefiltered EOG channels [11,12]. These classical detectors typically reported sensitivities of 75–88% with false-positive rates around 10–25% in semi-controlled EOG recordings [19]. Template-matching methods such as Boukadoum and Ktonas [16] improved temporal localization of individual saccades, yet over-segmentation remained common due to morphological overlap between blink slopes and saccadic onsets during REM sleep.

More recent work has revisited these threshold-based strategies in broader EOG applications, including wearable systems, free-viewing tasks and blink/saccade interfaces. Amplitude- or derivative-based thresholding combined with simple duration constraints achieves sensitivities of 80–95% in controlled settings, though often with task-dependent false-positive rates [36,37]. Deterministic velocity- and acceleration-threshold algorithms such as SaFiDe [38] generalize well across video, scleral coil and EOG traces, demonstrating robust segmentation of saccades and fixations without supervised learning. Similarly, recent quantitative evaluations of EOG eye-tracking show that velocity-threshold detectors can approach video-based performance under favourable signal-to-noise conditions [39].

Wavelet-based detectors using Haar or DB4 bases [14,15] improved transient localization compared to pure thresholds, but their symmetric wavelet shape lacks REM-specific priors and limits specificity in full-night PSG, where blinks, micro-saccades and drift artifacts frequently overlap in time. Collectively, these results indicate that while threshold and wavelet methods remain effective for short, controlled tasks, their generalization to realistic REM-rich overnight recordings is constrained by baseline variability, cross-event morphology and overlapping transients.

Recent work has shifted from rule-based detectors to machine learning and deep neural classifiers capable of distinguishing blinks, saccades, and gaze patterns directly from EOG. Classical feature-based pipelines on 1D EOG already achieve multi-class accuracies around 85–92% in human–computer interaction settings [40,41]. More recent hybrids that transform EOG into image-like representations (e.g., Irisgrams) and combine convolutional feature extraction with SVM decision layers report test accuracies up to approximately 96–99% for blink and eye-movement classification on small, controlled datasets [42]. Beyond isolated micro-events, several studies have demonstrated that EOG carries informative features for sleep staging: deep networks trained on single-channel EOG can reach overall accuracies of 76–85% on full-night PSG [43], and adding carefully designed EOG movement features to EEG-based models significantly improves N1 and REM F1-scores (e.g., REM from approximately 78% to approximately 84%) [44]. Further, small but consistent performance gains are observed when EOG is fused with optimized EEG montages [45]. However, most of these models still operate on coarse hypnogram-level classes and have not been validated against high-resolution REM microstructure, where the precise morphology of saccades, baseline drift, and pronounced inter-subject variability play a critical role.

In comparison, the proposed morphology-aware segmentation achieved 92.9% correct detections directly in full-night REM sleep, exceeding performance reported for previous REM-specific segmentation systems and approaching the upper range of detectors tested under ideal conditions. The custom kernel therefore provides a physiological advantage: it encodes prior knowledge about true saccadic morphology into the segmentation process, enabling robust and selective detection in signal conditions typical of clinical PSG. The modular design of the segmentation pipeline also allows the kernel to be replaced for other biosignals (e.g., EEG transients commonly analyzed with DB4 wavelets), extending the general applicability of the method.

### 4.2. Role of the SVM Classifier

Following segmentation, each candidate event was classified using a supervised SVM trained solely on EyeCon ground-truth recordings. The classifier relies on a compact feature vector consisting of (i) the amplitude–duration ratio, reflecting the well-established saccadic main-sequence relationship [33], and (ii) correlation with direction-specific log-sigmoid templates capturing typical rising and falling saccadic profiles. These features depend primarily on intrinsic physiological properties rather than absolute signal amplitude or device-specific noise, making them suitable for cross-dataset generalization.

A unique property of the present classifier is that it was trained and validated exclusively on EyeCon data, yet applied—without any retraining, recalibration, or domain adaptation—to full-night clinical PSG recordings. In contrast, many existing EOG-based classifiers are trained and tested on the same dataset or even the same recording session [17,19,20], resulting in inflated performance estimates and limited real-world robustness.

Previous controlled-environment studies reported high classification accuracies (e.g., 90–97% in [17]; 93–95% in [18]), but these evaluations were conducted under stable illumination, minimal head movement, and limited drift. When applied directly to PSG, morphology-based classifiers often show lower accuracy (typically 70–85%) due to drift, asymmetric blink contamination, and electrode instability across the night [15].

Despite these challenges, the proposed classifier achieved stable cross-domain behavior: 91.4% precision and 88.7% recall on EyeCon (Table 4), and consistent discrimination between saccades, blinks, and noise when applied to PSG recordings. Three factors likely contributed to this robustness: (i) morphology-based correlations vary little across hardware platforms; (ii) the low-dimensional feature space reduces overfitting and domain sensitivity; and (iii) training on EyeCon prevented the classifier from internalizing PSG-specific drift and blink artifacts. Taken together, these observations indicate that the classifier generalizes across hardware, recording conditions, and subject behavior—an essential requirement for large-scale REM microstructure research.

### 4.3. Interpretation of REM Microstructure

The combination of morphology-aware segmentation and cross-dataset SVM classification enabled full-night reconstruction of phasic and tonic REM for each participant. Phasic REM accounted for 31.8±3.5% of total REM duration, while tonic REM comprised 68.2±3.5%. These values fall within the physiological range reported for healthy adults, where phasic REM typically constitutes 20–35% of total REM sleep [1,10,15]. The observed phasic-to-tonic ratio (0.47±0.08) likewise aligns with prior findings that tonic REM generally dominates the microstructure [1,2].

The relatively low inter-subject variability (SD ≈ 3.5%) further highlights the stability of the proposed pipeline, despite the substantial variability in EOG amplitude, REM density, and recording quality across subjects. Manual scoring and threshold-based detectors typically yield much higher variability, as scorer fatigue leads to underestimation of phasic periods, while amplitude- or velocity-based detectors often overestimate phasic REM due to blink-related slopes and drift misclassified as bursts [1,10]. By relying on morphology-verified saccades and a cross-dataset classifier, the proposed system reduces these sources of bias and provides a more consistent quantitative readout of REM microstructure.

Physiological plausibility was further supported by EEG analysis, which showed increased beta and gamma spectral power during phasic REM—consistent with heightened cortical activation reported in prior neurophysiological studies [5,6]. Together, these findings support the utility of the method for large-scale REM microstructure analyses and for examining the role of phasic REM in emotional processing, autonomic regulation, and dream generation.

### 4.4. Comparison with Existing Literature

Prior work has shown that phasic REM is associated with increased cortical activation, limbic engagement, PGO-like activity, and autonomic fluctuations [1,5]. Abnormal phasic REM density has also been linked to depression, PTSD, REM sleep behavior disorder, and neurodegenerative conditions [1,15]. However, most earlier studies relied on manual inspection or on simple detection thresholds, limiting both scalability and reproducibility.

Compared with these approaches, the present study provides three major methodological advances: (i) a segmentation framework that uses a physiology-informed kernel tailored to real saccadic morphology, resulting in superior event delineation in PSG; (ii) a compact and interpretable SVM classifier trained entirely on an external dataset, thereby improving robustness and avoiding dataset-specific bias; and (iii) full-night phasic–tonic REM reconstruction validated against physiological norms and independent EEG markers. Together, these contributions bridge the gap between classical REM density estimation [10] and modern morphology-aware REM microstructure analysis, enabling reproducible large-scale studies.

### 4.5. Methodological Considerations

Although the proposed framework performs robustly, several methodological aspects merit consideration. First, the use of a single horizontal EOG channel limits the ability to analyze binocular direction or vertical saccades, and signal quality may degrade due to electrode drift or detachment later in the night. Nonetheless, maintaining single-channel processing avoids the propagation of artifacts across channels and preserves high temporal fidelity.Historically, high-precision eye-movement measurements have relied on scleral search-coil techniques, which are considered a laboratory gold standard for eye tracking [46]. However, such invasive or laboratory-bound methods are impractical for overnight polysomnography and incompatible with routine clinical sleep recordings, motivating the use of EOG-based approaches in this study.

Second, morphology-based detection depends on the choice of template. While the custom kernel used here improves generalization to PSG conditions, alternative kernels (e.g., DB4 or Symmlet wavelets) may be required for other biosignals such as EEG or EMG [27]. The modularity of the pipeline, however, allows for the straightforward substitution of alternative morphology templates.

Third, the use of an SVM with a low-dimensional feature space provides favorable robustness and computational efficiency, but more complex models—such as convolutional neural networks—may capture additional nuances of saccadic morphology at the cost of increased data requirements and reduced interpretability.

Finally, no PSG segment was used for training or optimization. While this ensures strong external validity, additional training datasets with PSG-specific ground truth could further improve performance, particularly for rare or ambiguous events.

### 4.6. Limitations

Several limitations should be acknowledged. First, the absence of ground-truth saccade labels in PSG recordings prevents direct computation of classification metrics on PSG data; instead, validation relied on microstructure plausibility and expert spot-checking. Second, horizontal-only EOG recording restricts the analysis to approximate saccadic magnitude rather than full two-dimensional eye movements. Third, the method’s performance depends on the quality of preprocessing, particularly drift removal and normalization, which vary across institutions and hardware. Fourth, while the custom kernel improves PSG specificity, it is tuned to the EyeCon-PSG combination and may require adaptation for datasets with substantially different electrode placements or sampling properties. Finally, the current pipeline does not incorporate adaptive drift compensation across multiple hours of recording, which may be beneficial for extremely long or artifact-heavy datasets.

### 4.7. Strengths and Implications

Despite these limitations, the proposed framework offers several strengths. It provides a transparent, interpretable, and computationally lightweight method that scales well to full-night recordings. Its morphology-aware segmentation, cross-dataset classifier, and modular design allow broad applicability across research settings and potential clinical use. By enabling reproducible extraction of phasic–tonic REM microstructure from routine PSG, the method lays a foundation for future studies on REM physiology, sleep biomarkers, emotional memory consolidation, and neuropsychiatric disorders.

## 5. Conclusions

This study introduced a fully automated and morphology-aware framework for detecting saccadic eye movements and reconstructing the phasic–tonic microstructure of REM sleep using a single clinical-grade EOG channel. The system combines a hybrid adaptive segmentation method—integrating MAD-based amplitude–change detection with a custom morphology kernel derived from manually verified saccades—with a compact, physiologically interpretable SVM classifier.

All components were trained exclusively on the publicly available EyeCon dataset and subsequently applied, without modification, to full-night clinical PSG recordings. This strict separation between training and validation ensured that the evaluation on PSG represented genuine external validation rather than dataset-specific tuning.

Across 21 overnight PSG recordings, the method demonstrated strong agreement with expert visual scoring, achieving 92.9% correct detections, 5.3% fragmentation, and only 1.8% missed events. By integrating morphology-aware segmentation with a robust, low-dimensional classifier, the proposed pipeline provides a transparent, computationally efficient, and clinically meaningful tool for REM microstructure analysis.

Its modular architecture also enables straightforward adaptation to other biosignals or multimodal extensions, making it suitable for large-scale sleep research and automated sleep phenotyping. Beyond retrospective PSG analysis, the framework lays a solid foundation for future work investigating the diagnostic and mechanistic significance of phasic REM in neuropsychiatric disorders, cognitive–emotional processing, and brain–body dynamics during sleep.

## Figures and Tables

**Table 1:** Summary of key hyperparameters and implementation settings used in the proposed segmentation and classification framework.

Processing Stage	Parameter	Description
Preprocessing	Baseline removal window	Moving-average filter applied within each REM interval
Preprocessing	Band-pass filtering	2nd-order zero-phase Butterworth filter (1–10 Hz)
Normalization	Z-score reference interval	Computed separately for each expert-scored REM interval
Amplitude-based score	Derivative definition	Absolute first derivative |x(t)−x(t−1)|
Amplitude-based score	MAD scaling factor $k_{\mathrm{MAD}}$	Optimized using EyeCon ground-truth data
Morphology score	Kernel type	Custom saccade-derived morphology kernel
Morphology score	Kernel normalization	L2-normalized to unit energy
Score fusion	Fusion weight α	Controls balance between amplitude- and morphology-based scores
Boundary detection	Thresholding rule	Mean + 3 standard deviations (per REM interval)
Boundary refinement	Minimum segment duration min_dur	Prevents detection of sub-physiological segments
Boundary refinement	Merging gap *g*	Merges temporally adjacent segments
Segment classification	Classifier type	Support vector machine (RBF kernel)
Segment classification	Feature set	Amplitude–duration ratio and morphology-based correlation
Segment classification	Training data	Balanced EyeCon segments (saccade, blink, artifact)

**Table 2:** Mapping of EyeCon and clinical PSG datasets onto the functional components of the proposed processing pipeline.

Pipeline Component (Block)	EyeCon	Clinical PSG
*1. Dataset Acquisition and REM Interval Extraction*		
Dataset acquisition	✔	✔
REM interval extraction (AASM scoring)	—	✔
*2. Preprocessing*		
Baseline drift removal, filtering, normalization	✔	✔
Preprocessing parameter validation	✔	—
*3. Hybrid Adaptive Segmentation*		
Amplitude and morphology score computation	✔	✔
Hybrid score fusion and boundary detection	✔	✔
Boundary refinement	✔	✔
Segmentation hyperparameter optimization (Bayesian)	✔	—
Morphology kernel construction (template creation)	✔	—
*4. Feature Extraction*		
Feature extraction (segment-level)	✔	✔
Feature behavior calibration	✔	—
*5. Segment Classification Using SVM*		
SVM-based segment classification (inference)	✔	✔
Training dataset preparation (balanced classes)	✔	—
SVM parameter optimization (Bayesian)	✔	—
*6. Phasic and Tonic REM Reconstruction*		
Saccade grouping, burst formation	—	✔
Phasic/tonic REM reconstruction	—	✔
*7. Evaluation*		
Segmentation/classification metrics (with ground truth)	✔	—
REM microstructure evaluation (physiological plausibility)	—	✔
Temporal accuracy, fragmentation, merging analysis	✔	—

**Table 3:** Segmentation accuracy on EyeCon (mean ± SD).

Metric	Mean	SD
Correct detections (%)	92.9	2.6
Fragmented (%)	5.3	1.0
Missed events (%)	1.8	1.8
Detected/GT ratio	1.59	0.11

**Table 4:** Saccade classification accuracy on EyeCon (mean ± SD).

Metric	Mean	SD
Precision (%)	91.4	3.8
Recall (%)	88.7	4.2
Specificity (%)	96.1	1.9
F1-score	0.900	0.021
Cohen’s κ	0.76	0.04

**Table 5:** Phasic and tonic REM values across the 21-subject PSG cohort. Values are mean ± SD.

Parameter	Mean	SD
**Durations (minutes)**		
Phasic REM duration	30.6	10.0
Tonic REM duration	64.9	18.9
Total REM duration	95.5	27.8
**Proportions**		
Phasic REM (%)	31.8	3.5
Tonic REM (%)	68.2	3.5
Phasic/Tonic ratio	0.471	0.079

## Data Availability

Clinical PSG data cannot be publicly shared due to GDPR and institutional data-protection regulations. Fully anonymized versions of these recordings may be provided upon reasonable request to the corresponding author and are subject to institutional approval. Derived segmentation outputs produced in this study are likewise available upon request. All processing scripts, including the complete segmentation and classification pipeline and accompanying documentation, are publicly accessible on GitHub at: https://github.com/Tomas-Nagy-BiomTech/Automated-Detection-of-Phasic-REM-from-EOG.git (accessed on 12 February 2026).

## Abbreviations

REM	Rapid Eye Movement
EOG	Electrooculography
PSG	Polysomnography
EEG	Electroencephalography
MAD	Median Absolute Deviation
DB4	Daubechies-4 Wavelet
SNR	Signal-to-Noise Ratio
SVM	Support Vector Machine
RBF	Radial Basis Function
GT	Ground Truth
ROC	Receiver Operating Characteristic
F1	F1-score (Harmonic Mean of Precision and Recall)
AASM	American Academy of Sleep Medicine
HFO	High-Frequency Oscillation
LSL	LabStreamingLayer
